# Clinical Outcome Assessments in Implant Dentistry Clinical Research

**DOI:** 10.1111/clr.70066

**Published:** 2026-02-24

**Authors:** Muhammad H. A. Saleh, Frank Schwarz, Hom‐Lay Wang, Ronald E. Jung, John H. Powers

**Affiliations:** ^1^ Department of Periodontics and Oral Medicine, School of Dentistry University of Michigan Ann Arbor Michigan USA; ^2^ Department of Oral Surgery, Implantology and Oral Medicine Goethe University Frankfurt Germany; ^3^ Center of Dental Medicine, Clinic of Reconstructive Dentistry University of Zürich Zürich Switzerland; ^4^ Department of Medicine George Washington University School of Medicine Washington DC USA

## Abstract

Precise terminology and a clear distinction between what is measured (outcomes) and how it is measured (outcome assessments) are fundamental in implant dentistry (ID) research. This narrative review defines outcomes and outcome assessments and aligns ID terminology with established regulatory frameworks. We map survival outcomes, biomarkers, and the four FDA‐recognized clinical outcome assessments (COAs) patient‐reported, clinician‐reported, observer‐reported, and performance outcomes onto a spectrum defined by patient meaningfulness and the need for human judgment. Definitive events (e.g., implant loss) sit at one end of the spectrum, and purely objective biomarkers (e.g., dimensional ridge changes) sit at the other end, while COAs occupy the middle of that spectrum. Content validity is essential for any COA. Tools must be co‐developed with people with lived experience (PWLE) and evaluated with Consensus‐based Standards for the selection of health Measurement INstruments (COSMIN) standards to ensure they truly measure the intended construct, in the right population, with adequate reliability, responsiveness, and interpretability. Finally, we caution against conflating measures (tools, e.g., radiography) with endpoints (prespecified analysis variables with time frame and threshold). Using standardized definitions in present and future ID consensus statements will increase their meaningfulness and strengthen the relevance and comparability of clinical trials.

## 1 Introduction

A key aspect of research and clinical practice in implant dentistry (ID) is understanding the benefits and harms of interventions on patient outcomes. An imperative yet simple principle for a *valid and meaningful outcome assessment* is anchored in a concept of interest that matters to people: survival, how they feel, or how they function in their daily lives (Powers 3rd et al. 2017). All other measures are proxies on a spectrum of evidentiary probability.

In regulatory and research contexts, clinical outcomes are classified in a structured way, often termed endpoints, with precise definitions and measurement methods. For example, the U.S. Food and Drug Administration (FDA) defines *clinical outcome assessments* (*COAs*) as a specific measure of how a patient *feels*, *functions*, *or survives*. Clinical trials designate primary endpoints (the main outcome assessments of interest) and secondary endpoints, relying on validated metrics to quantify benefit.

Doctors in everyday practice use simpler, more flexible criteria to gauge success, rather than rigid numerical scales for every patient. Though crucial, detailed outcome measures from trials (e.g., lengthy questionnaires or scoring systems) are unfortunately not routinely tracked for every patient, partly because of the extra time/effort, as well as the unclear immediate benefit to patient management.

Recently, the International Society for Pharmacoeconomics and Outcomes Research (ISPOR) Clinical Outcomes Assessment Task Force developed two task force reports covering definitions and emerging good measurement practices for Clinician‐Reported Outcomes (ClinROs) assessment development and evaluation (Powers 3rd et al. 2017; Coyne and Wyrwich 2015; Walton et al. 2015; Benjamin et al. 2017). In this commentary, we outline how each category of outcome assessment can be practically applied to clinical research *and* everyday decision‐making in ID.

## 2 Definitions

*Outcomes* refer to the concepts that are evaluated.

*Outcome assessments* refer to the actual measures used to perform the evaluation. This includes who (or what) performs the measurements and how they are performed. For instance, pain as a concept can be measured by several different pain scales, which are the actual outcome assessments. Subjective concepts can be objectively measured if appropriate measures are developed, measured, and interpreted properly. Appropriate outcome assessments may include both COAs and biomarkers.

A *valid* outcome assessment means that the outcome measures what it purports to measure. The concept of “true outcome” is complex, since all outcome measures have some degree of error.

A *meaningful* outcome assessment means it matters to patients, which, along with validity, are the two most important characteristics.

A *clinical benefit* is a clinically meaningful positive effect of an intervention, i.e., a positive effect on how an individual feels, functions, or survives.

*Endpoints* are how outcome assessments are used in the setting of a clinical research study, which includes the hierarchy of outcome assessments (how they are combined or ranked), the timing of measurements, statistical testing (difference in proportions or time to event), and interpretation of results (how much difference makes a difference to patients).

*Composite endpoints* combine more than one measure into a single endpoint, but one can also evaluate co‐primary endpoints where two or more outcomes are evaluated as separate endpoints. An example of that in ID is peri‐implantitis, whose case definition is a combination of (i) clinical signs of bleeding or suppuration, (ii) probing depth increase, and (iii) radiographic evidence of bone loss (Berglundh et al. 2018; Papapanou et al. 2018).

A *Measure* is the tool used to evaluate the concept (the outcome). A measure becomes an endpoint when *measurement timing* is prespecified, analysis is planned to test a prespecified hypothesis, and *results* are interpretable for patient benefit.

*Biomarkers* are defined as a characteristic that is measured as an indicator of normal biological processes, pathogenic processes, or biological responses to an exposure or intervention, including therapeutic interventions. This includes histological or radiographic characteristics.

Patient‐Reported Outcomes (PROs): Are measurements based on reports that came directly from the patient without interpretation of the patient’s response by a clinician or anyone else. Symptoms or other concepts known only to the patient can only be measured by PRO measures.

*Clinician‐Reported Outcomes* (*ClinROs*): Here, a member of the investigator team (e.g., a clinician other than the primary investigator) is the rater. This member's own professional training and judgment are relied upon to provide a score. These outcomes reflect the clinician's expert observation of the patient's health status, rather than a direct measurement of a biological parameter.

*Observer‐Reported Outcomes* (*ObsROs*): Observations are recorded according to the judgment of a person (observer) who does not require specialized professional training.

*Performance Outcomes* (*PerfOs*): The patient is assessed by performing a defined task that is quantified in a specified way (by the investigator or other calibrated personnel). Yet, the investigator does not apply judgment to quantify the performance.

## 3 Structure of Different Outcomes

*Outcomes* span a continuum defined by two axes: Subjectivity of the concepts (the need for human judgment) and direct patient meaningfulness (whether the concept captures how a patient feels, functions, or survives) (Powers 3rd et al. 2017).

**FIGURE 1:**
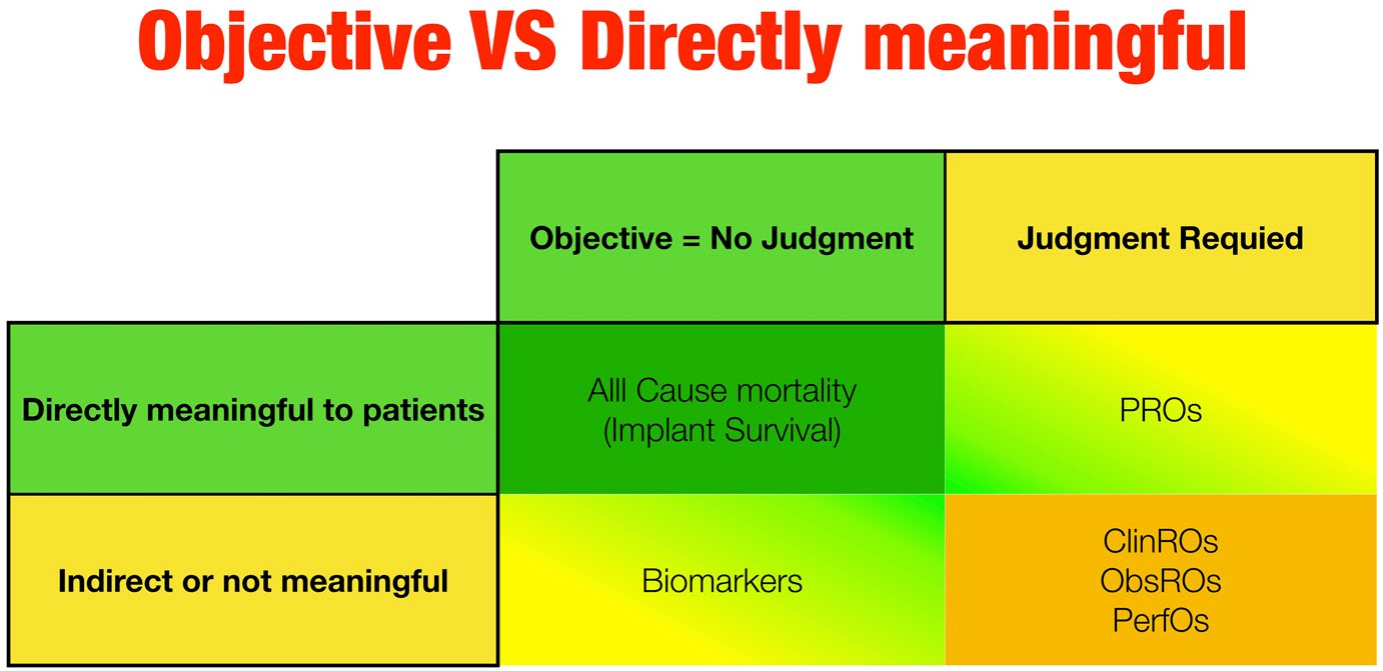
A Classification of outcome measures by meaningfulness to patients and objectivity of the outcome. ClinROs, Clinician‐Reported Outcomes; ObsROs, Observer‐Reported Outcomes; PerfOs, Performance Outcomes; PROs, Patient‐Reported Outcomes.

At one end of the spectrum of outcomes are “hard/definitive” events—e.g., all‐cause mortality, implant or tooth loss—which require no interpretive judgment and intrinsically matter to patients (Figure 1).

In the medical field, when *survival* outcomes are used as endpoints, they often record time to death or survival rate at a fixed time point. In ID this is somewhat synonymous with implant loss or tooth loss. These are *direct* measures of ultimate patient benefit, i.e., no interpretation or judgment is required.

*Biomarkers* are outcome assessments that are not influenced by the patient's motivation or volition or a rater's judgment. This includes biochemical measurements of blood, blood pressure measurements, and quantitative measurements of radiographic images. Biomarkers, such as three‐dimensional ridge changes or inflammatory cytokine levels, are objective concepts yet fall short of meaningfulness; a 10‐mmHg blood‐pressure reduction or a 1 mm bone gain is not a direct measure of patient comfort or function on its own (Guidance for Industry: FDA 2006).

A biomarker can become clinically *meaningful* only when it is validated as a *surrogate endpoint*, i.e., when a clear mechanistic rationale and strong clinical data show that changes in the biomarker reliably predict a specific patient‐relevant *clinical benefit.* However, more often, the biomarker is used specifically because it might reflect a direct future benefit for patients. The reason to use a biomarker is often that it can be measured earlier in time than the direct patient outcome. Biomarkers require thorough validation to ensure that the test, tool, or instrument is adequate for its proposed use.

On the other spectrum lie *Patient‐Reported Outcomes* (*PROs*) concepts. These concepts are “concepts” that are known only to patients and for which the report comes directly from the patient and is recorded without interpretation by anyone else. PROs like post‐surgical pain or denture satisfaction directly reflect the patient's lived experience but are subjective concepts. However, these subjective concepts can be measured objectively if PRO measures (PROMs) are developed, measured, and interpreted properly.

Between Biomarkers and PROs lie other outcomes, namely the FDA‐defined COAs: clinician‐, observer‐, and performance outcomes (ClinROs, ObsROs, PerfOs) (Powers 3rd et al. 2017). These measures incorporate human judgment in their measurement, just like PROs. Yet, unlike PROs, they act as indirect surrogates for patient benefit.

In chronic oral conditions—where definitive events (e.g., mortality) are rare—assessing *biomarkers* with complementary use of these COAs is essential to capture a full, patient‐centered picture of treatment effects (Powers 3rd et al. 2017). The FDA‐NIH BEST Resource (*BEST* [*Biomarkers, EndpointS, and Other Tools*] *Resource* 2016) categorizes biomarkers by their intended use and acknowledges that only a subset (e.g., fully validated response biomarkers) currently meets the evidentiary bar to serve as surrogate endpoints linked to patient benefit. Most biomarkers remain “candidate” or “reasonably likely” surrogates that require rigorous trial‐level validation to confirm that biomarker changes reliably predict meaningful improvements in patient‐centered outcomes.

## 4 Outcome Assessments as Endpoints

### 4.1 Survival Outcomes as Endpoints

4.1.1

In the medical field, all‐cause mortality is the “cleanest possible endpoint” because it is unambiguous and universally meaningful. Tooth loss due to any reason (Overall Tooth Loss [OTL]) or Overall Implant Loss (OIL) are considered “survival outcomes”. By analogy, a 5‐year overall survival in colon cancer can translate in ID to a 5‐year implant survival. Listing tooth‐loss or implant‐loss specific causes (e.g., tooth loss due to unrestorability) turns this metric into a ClinRO because assigning the cause relies on professional judgment (see more below on ClinROs).

4.1.2

4.1.2.1

*Strengths*: No translation regarding meaningfulness to patients is needed, unlike biomarkers. It should be a valid and meaningful endpoint for implant and tooth therapy.

*Limitations*: It requires long‐term follow‐up and can be insensitive to functional or symptomatic changes in chronic conditions. On the flip side, this means that measuring symptoms and function is also useful when using survival outcomes.

### 4.2 Biomarkers as Endpoints

The BEST resource (*BEST* [*Biomarkers, EndpointS, and Other Tools*] *Resource* 2016) outlines seven non‐overlapping biomarker categories that define different uses for biomarkers (e.g., diagnosing disease in contrast to outcomes). Biomarkers can be categorized as: *diagnostic* (confirm disease presence), *monitoring* (track status over time), *predictive* (indicate how response to therapy modifies the treatment effect and require a treatment‐by‐marker interaction), *prognostic* (forecast natural history independent of treatment), *response* (demonstrate biological change after exposure to a medical product or therapy), *safety* (biomarkers indicate the likelihood, presence, or extent of toxicity), and *susceptibility/Risk* (indicates an individual's likelihood of developing a disease in the future, before clinical onset). A *multicomponent biomarker* is an algorithmic composite of several biomarkers. Multicomponent biomarkers that include any ClinROs should be considered ClinRO (e.g., The Implant Disease Risk Assessment (IDRA) tool) (Heitz‐Mayfield et al. 2020).

Unlike survival and PROs, biomarkers do not tell us how a patient with a dental implant feels, functions, or whether the implant will survive. Instead, they capture biological characteristics that may predict or mediate patient outcomes (Cagney et al. 2018). Biomarker acquisition and interpretation require training and calibration, but do not involve subjective clinical judgment.

### 4.3 Clinical Outcome Assessments (COAs) as Endpoints

COAs are instruments that *directly* or *indirectly* measure how patients *feel or function*. The FDA recognizes four COA categories: PRO (*direct assessment*), ClinRO, ObsRO, and PerfO (*indirect assessments*). Unlike biomarkers, which require no subjectivity in their assessment, COAs rely on the implementation, interpretation, and/or reporting from a patient, a clinician, or an observer (Coyne and Wyrwich 2015; Walton et al. 2015). Below each is described with brief considerations.

#### 4.3.1 Patient‐Reported Outcomes (PROs)

PROs are *self‐reported* concepts without amendment by clinicians or anyone else. A *patient‐reported outcome measure* (PROM) is a tool developed to measure the concept of PRO (Table 1).

**TABLE 1:** Examples for PROs use‐case in medicine with examples relevant to ID.

Concept (PRO)	Tool (PROM)
Acute symptom severity	Visual Analog Scale (VAS) after ARA surgery (Wortmann et al. 2019)
Function	Oral Health Impact Profile‐14 (OHIP‐14) to capture chewing ability (Yunus et al. 2014)
Health‐related quality of life	Patient's Satisfaction and Change of Psychology (Wu et al. 2015) questionnaire

#### 4.3.2 Clinician‐Reported Outcomes (ClinROs)

A ClinRO requires specialized training in its evaluation. Each ClinRO starts with a clinical procedure that yields either a reading, a rating or a global assessment. During that procedure, the patient may remain passive (e.g., seated calmly), take an active role (e.g., perform a requested movement), or answer targeted questions about symptoms or daily activities that are interpreted by clinicians (Powers 3rd et al. 2017).

ClinROs are judgment‐based ratings by trained health professionals. They can be split into three groups: (1) *ClinRO reading*: is reported in a dichotomous (e.g., yes/no) form (e.g., presence or absence of root fracture), (2) *ClinRO rating*: characteristics observed and reported have at least 3 categories of interest (e.g., Implant Disease Risk Assessment, IDRA), and (3) *ClinRO clinician global assessments*: based on overall judgment (e.g., is the patient better or worse, ability of patient to clean as judged by the clinician, overall periodontal stability, need for additional grafting, or need for prescribing systemic antibiotics following a surgical complication). The clinician then interprets these observations to generate the final outcome, unlike patient‐reported outcomes, which do not involve clinician interpretation (Mortimer 2007).

If specific causes of tooth or implant loss such as tooth loss due to periodontitis (TLP) or implant loss due to peri‐implantitis (ILP) are to be reported, they should be considered as ClinROs since they often require judgment regarding the specific cause of TLP or ILP (Table 2).

**TABLE 2:** Examples for ClinROs in medicine with examples relevant to ID.

Example setting	ClinROs example	Notes
Solid tumors	Response Evaluation Criteria in Solid Tumors (RECIST 1.1) (Andre et al. 2008)	Requires quantitative imaging, but clinician interpretation is still required.
Implant dentistry	Bleeding on probing/suppuration	This is an example of *ClinRO reading*. After probing, the presence of bleeding or suppuration is reported in a dichotomous (e.g., yes/no) form.
	Pink Esthetic Score (PES) (Furhauser et al. 2005), White Esthetic Score (WES) (Belser et al. 2009)	This is an example of *ClinRO rating.* Multiple assessments relying on clinicians' judgment
	The need for prescribing a systemic antibiotic following graft infection (Castagna et al. 2013)	This is an example of *ClinRO global assessment.* Variables assessed in clinician's decision are poorly defined
	Implant loss due to peri‐implantitis	This relies on clinicians' judgment and expertise in treating peri‐implant diseases

#### 4.3.3 Observer‐Reported Outcomes (ObsROs)

ObsROs are reported observations by nonclinical observers (family, caregivers, teachers) for patients unable to self‐report (Benjamin et al. 2017). These are not “delegate reports”. Rather, they rely on direct observations of signs or behaviors (Table 3). An example in patients with dementia that demonstrated high sensitivity in detecting cognitive impairment compared to other informant‐based dementia screening tools is the eight‐item interview to ascertain dementia (AD8) (Taylor‐Rowan et al. 2023).

**TABLE 3:** Examples for ObsROs in medicine with examples relevant to ID.

Example setting	ObsRO example
Asthma	Parent‐proxy cough‐specific quality of life [PC‐QOL] (Chang et al. 2012)
Dentistry	Symmetric alteration index (Kokich et al. 2006)

#### 4.3.4 Performance Outcomes (PerfOs)

Standardized tasks are administered to patients; the outcome is quantitative performance. Unlike PROs or ClinROs, PerfOs can be device‐recorded but still require protocolized administration and patient effort of involvement in performing the test (Table 4).

**TABLE 4:** Example of PerfOs in medicine with an example relevant to ID.

Example setting	PerfO example
Mobility	6‐Minute Walk Test (6MWT) in COPD (Beaumont et al. 2019)
Dental implants	The Masticatory performance test (Slagter et al. 1993; Stellingsma et al. 2005)

## 5 Content Validity

While there is a need to create dental COAs of all domains (PROs, ClinROs, ObsROs, and PerfOs), content validity must be ensured for any utilized or created COAs (Patrick et al. 2011). Unlike medical disciplines, very few ID‐specific COAs exist, and even fewer meet the FDA qualification standards. Engaging people with lived experience (PWLE), like patients who have undergone dental implants (yielding PROs) and their carers (yielding ObsROs), ensures measures capture what truly matters to patients and carers. Involving them in developing guidance (not just applying it) aligns research endpoints with real‐world priorities and improves health literacy and adherence (Needleman 2014; Needleman et al. 2023).

The Consensus‐based Standards for the selection of health Measurement INstruments (COSMIN) initiative provides a consensus‐based taxonomy, terminology, and set of methodological standards for evaluating the measurement properties of COAs, including content validity as well as other measurement properties (Mokkink et al. 2006). By systematically grading evidence on content validity, structural validity, reliability, measurement error, cross‐cultural validity, criterion and construct validity, and responsiveness, the COSMIN tools help determine whether a given COA truly measures the construct it claims to measure, in the intended population and context, with sufficient precision and sensitivity to detect meaningful change (Terwee et al. 2018).

The recently published Implant Dentistry Core Outcome Set and Measurement (ID‐COSM) initiative developed a core set of mandatory outcomes for clinical trials in ID and/or soft tissue/bone augmentation (Tonetti, Heitz‐Mayfield, et al. 2023; Tonetti, Sanz, et al. 2023). ID‐COSM followed the COMET framework to establish “what” should be measured in every implant study, using a rigorous, multi‐stakeholder Delphi process (patients, clinicians, methodologists, industry) (Williamson and Clarke 2012). For that, it specifically followed the Outcome Measures in Rheumatoid Arthritis Clinical Trials (OMERACT) (Tugwell and Boers 1993) established framework.

Many outcomes reported in the dental literature still default to “heritage variables” (i.e., precedent outcomes used in prior studies) instead of first asking whether those variables really matter to patients. Investigators often elevate laboratory surrogates and clinician ratings simply because “that's how it's always been done.” A better hierarchy starts with outcomes that change a person's life or longevity (implant or tooth survival, and pain‐free function) and only then layers in explanatory biomarkers and other COAs that clarify mechanisms or safety. COSMIN then makes sure whatever instruments are chosen for these layers are valid, reliable, and responsive—i.e., address “how” to measure those outcomes. The same principle should govern lists of “mandatory” versus “optional” outcomes in new consensus statements. An endpoint deserves the *mandatory* label only if (i) its clinical or patient‐centered relevance has been demonstrated and (ii) the measurement tool meets COSMIN quality standards; otherwise, it belongs in the optional or exploratory outcomes.

**FIGURE 2:**
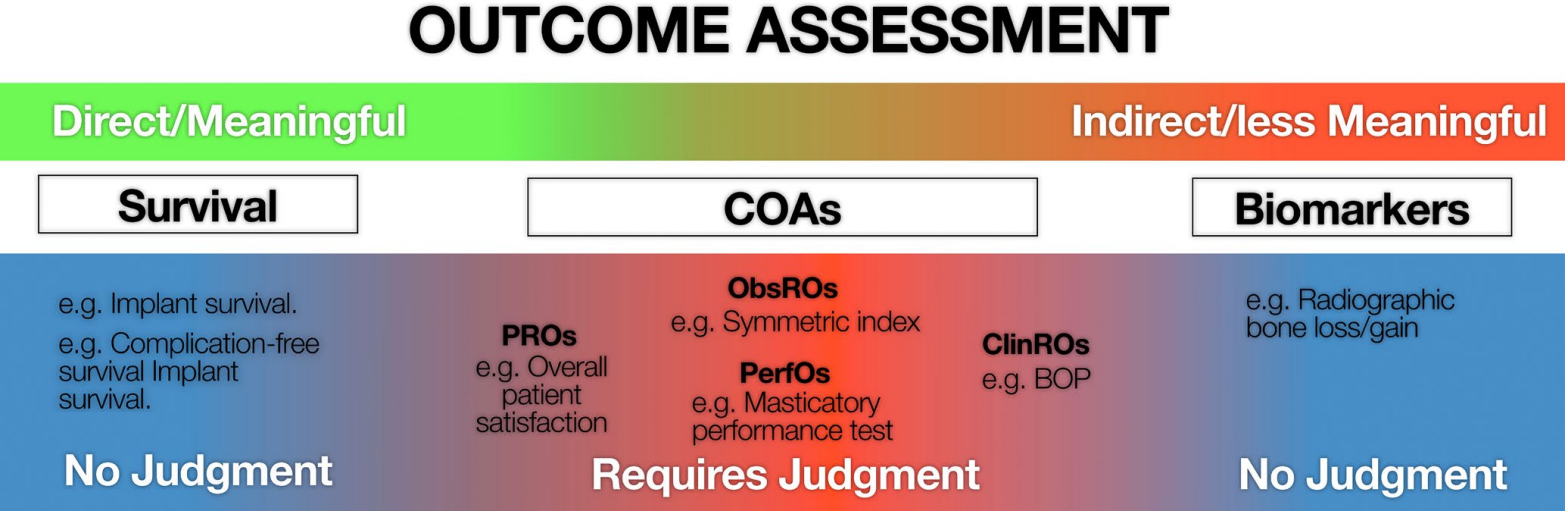
Examples of outcome assessments in implant dentistry.

Figure 2 shows how outcome assessments fall along one end, where fully objective survival endpoints lie, and at the opposite extreme are biomarkers, which likewise yield objective data without interpretive judgment but offer only indirect, surrogate information that does not directly reflect how patients feel or function. Between these lie clinical outcome assessments (COAs).

## 6 Common Misconceptions and Grey Zones

*Quantitative imaging is always a biomarker*: Only if it is purely quantitative and done with minimal clinician judgment. Determining root fracture as yes/no is considered a ClinRO. Determining horizontal bone gain using superimposed CBCT images is considered a biomarker. Peri‐implant radiographic bone loss measured on standardized radiographs is a biomarker.

*ClinROs replace PROs when patients cannot report*: True, but contingent on meaningfulness. There should be evidence that the ClinRO reflects direct outcomes that are meaningful to patients. ObsROs or PerfOs may be more appropriate in settings where a non‐trained observer can make the measurements; each is distinct with unique validation needs (Nair et al. 2023).

*Biomarkers are easier to validate*: Not necessarily. Analytical validity may be straightforward, but clinical validation (*does the biomarker predict effects of treatments showing patient benefit?*) is challenging. Patient‐level correlation (showing more or less of a biomarker is prognostic for a patient outcome) is not the same as trial‐level validation of treatment effects (showing that a treatment‐mediated change on a biomarker predicts and reflects a treatment‐mediated change on a direct patient outcome at the trial level).

*Outcome measures and endpoints are synonyms*: Not true. An outcome measure becomes an endpoint only when it has a predefined timing of measurements, is statistically analyzed to test a prespecified hypothesis, and is interpretable for patient benefit.

## 7 Conclusions

Demonstrating “how the patient is better off” in terms of living longer and living better should be our main trajectory. Survival, Biomarkers, and COAs form an ordered structure from direct clinical benefit to indirect biological signals. A single clinical trial can often employ multiple outcome assessments used as endpoints depending on the goals of the study: survival (provides a definite outcome), COAs (provides patient relevance), and biomarker (provides an earlier mechanistic insight), where the exact measurement can occupy different categories across studies.

Developing valid COAs is crucial for clinical research to determine the direct benefits and harms of interventions. This approach can also be extended to clinical practice to enhance diagnostic accuracy, determine which patients may benefit from treatments, and follow patients over time. Validating a COA for ID means co‐developing it with people with lived experience, confirming content validity (e.g., via COSMIN), and then proving its structural validity, reliability, responsiveness, and clear interpretability in implant‐specific populations.

## Author Contributions

**Muhammad H. A. Saleh:** conceptualization, investigation, writing – original draft. **Frank Schwarz:** writing – review and editing. **Hom‐Lay Wang:** writing – review and editing. **Ronald E. Jung:** writing – review and editing. **John H. Powers III:** project administration, supervision, writing – review and editing.

## Ethics Statement

The authors have nothing to report.

## Conflicts of Interest

The authors declare no conflicts of interest.

## Data Availability

The data that support the findings of this study are available at the request of the corresponding author. The data are not publicly available due to privacy or ethical restrictions.
